# Reply to Burgess et al: Catastrophic climate risks are neglected, plausible, and safe to study

**DOI:** 10.1073/pnas.2214884119

**Published:** 2022-10-10

**Authors:** Luke Kemp, Chi Xu, Joanna Depledge, Kristie L. Ebi, Goodwin Gibbins, Timothy A. Kohler, Johan Rockström, Marten Scheffer, Hans Joachim Schellnhuber, Will Steffen, Timothy M. Lenton

**Affiliations:** ^a^Centre for the Study of Existential Risk, University of Cambridge, Cambridge CB2 1SB, United Kingdom;; ^b^Darwin College, University of Cambridge, Cambridge CB3 9EU, United Kingdom;; ^c^School of Life Sciences, Nanjing University, Nanjing 210023, China;; ^d^Cambridge Centre for Environment, Energy and Natural Resource Governance, University of Cambridge, Cambridge CB2 3QZ, United Kingdom;; ^e^Center for Health and the Global Environment, University of Washington, Seattle, WA 98195;; ^f^Future of Humanity Institute, University of Oxford, Oxford OX2 0DJ, United Kingdom;; ^g^Department of Anthropology, Washington State University, Pullman, WA 99164-4910;; ^h^Santa Fe Institute, Santa Fe, NM 87501;; ^i^Cluster of Excellence ROOTS - Social, Environmental, and Cultural Connectivity in Past Societies, Christian-Albrechts-Universität, 24118 Kiel, Germany;; ^j^Potsdam Institute for Climate Impact Research, 14473 Potsdam, Germany;; ^k^Department of Environmental Sciences, University of Wageningen, 6708PB Wageningen, The Netherlands;; ^l^Earth System Science Department, Tsinghua University, Beijing 100190,China;; ^m^Fenner School of Environment and Society, The Australian National University, Canberra, ACT 2601, Australia;; ^n^Global Systems Institute, University of Exeter, Exeter EX4 4QE, United Kingdom

We thank Burgess et al. (1) for their contribution, “Catastrophic climate risks should be neither understated or overstated,” in response to “Climate Endgame” (2). We agree that studying catastrophic climate scenarios and extreme risk mitigation is imperative.

We disagree that catastrophic scenarios are already adequately or excessively studied. Counting the Shared Socioeconomic Pathway (SSP) SSP3-7.0 and SSP5-8.5 scenarios used in the Intergovernmental Panel on Climate Change AR6 Working Group II report is not a good proxy for catastrophic climate risk assessment. The scenarios only account for anthropogenic emissions (each with a range of temperature outcomes), not extreme risk assessment. Even just for high-end warming scenarios, more granular text-mining reports (3, 4) show these are under-studied relative to their likelihood and lower-warming scenarios. This is supported by literature sampling (3) and reflections by popular writers who have synthesized the climate risk literature (5, 6). The normally used cutoff date of 2100 in modeling also contributes to an underappreciation of long-term higher warming and catastrophic scenarios.

High-end warming scenarios are plausible. As we note in “Climate Endgame” (2), while anthropogenic emissions in line with SSP3-7.0 or SSP5-8.5 appear unlikely, temperatures consistent with these scenarios could be reached due to stronger than expected Earth system responses or after a longer duration of anthropogenic emissions. Moreover, we should not place undue confidence in long-term forecasts. Even groups of “superforecasters” cannot make accurate predictions of geopolitical events more than a year out (7).

Relying on projected likely economic damages from integrated assessment models is poor risk management due to their well-known limitations and flaws (8). Climate policy is better thought of as buying insurance against catastrophic outcomes (9).

Throughout their letter, Burgess et al. (1) conflate catastrophic climate scenarios with high-end warming scenarios: a common mistake. As we stress in “Climate Endgame” (2), lower levels of warming could result in catastrophic outcomes because overall risk is contingent on at least five factors:

1) Anthropogenic emissions;
2) Earth System responses;
3) Climate change impacts;
4) Societal fragility;
5) Human system responses.

Disaster could occur across any of these layers. Even if each layer were independent (and they are not) and we assume a 99% chance of each having a noncatastrophic outcome, then there is still a 5% chance of catastrophe across the entire chain.

Burgess et al. (1) warn that discussion of extreme risks could justify dangerous policies. Democracies require honest, open, and accurate scientific communication. Furthermore, understanding extreme risks does not automatically lend support to dictatorial responses. For instance, modeling of nuclear winter empowered bottom-up and multilateral disarmament efforts. Placatory misinformation can also lead to dangerous policy making. At present, no country has policies in place compatible with a 1.5 °C warming ceiling (10).

We wish to promote safe and inclusive risk management. This is why we suggested the use of deliberative democratic methods in “Climate Endgame” (2). These can be effective safeguards against dangerous responses (11).

A lack of attention to extreme risks or completely speculative doom mongering would more likely lead to maladaptive responses and mental health stresses than the informed deliberation over catastrophic risks that we propose.
